# Dynamics of the Gut Bacteria and Fungi Accompanying Low-Carbohydrate Diet-Induced Weight Loss in Overweight and Obese Adults

**DOI:** 10.3389/fnut.2022.846378

**Published:** 2022-02-11

**Authors:** Dan Yu, Libin Xie, Wei Chen, Jin Qin, Jingjing Zhang, Min Lei, Yue Wang, Hongge Tang, Sujuan Xue, Xinxiu Liang, Zelei Miao, Congmei Xiao, Meishuang Shang, Jie Lu, Hailing Di, Yuanqing Fu

**Affiliations:** ^1^Department of Nutrition, The Third Hospital of Hebei Medical University, Shijiazhuang, China; ^2^School of Chemical Engineering, Shijiazhuang University, Shijiazhuang, China; ^3^Department of Orthopedic Surgery, Third Hospital of Hebei Medical University, Shijiazhuang, China; ^4^The Biobank, Third Hospital of Hebei Medical University, Shijiazhuang, China; ^5^Hebei Orthopedic Clinical Research Center, Third Hospital of Hebei Medical University, Shijiazhuang, China; ^6^Clinical Biochemistry Lab, Third Hospital of Hebei Medical University, Shijiazhuang, China; ^7^Key Laboratory of Growth Regulation and Translational Research of Zhejiang Province, School of Life Sciences, Westlake University, Hangzhou, China; ^8^Westlake Intelligent Biomarker Discovery Lab, Westlake Laboratory of Life Sciences and Biomedicine, Hangzhou, China; ^9^Institute of Basic Medical Sciences, Westlake Institute for Advanced Study, Hangzhou, China

**Keywords:** low-carbohydrate diet, obesity, weight loss, gut bacteria, gut fungi

## Abstract

**Background:**

Low-carbohydrate (e.g., Atkins) dietary pattern is one of the most effective diets for weight loss, but little is known about the characteristics of the gut microbiota accompanying low-carbohydrate diets-induced weight loss. This study aims to profile dynamics of gut bacteria and fungi accompanying modified Atkins diets-induced weight loss among overweight and obese adults.

**Methods:**

Overweight and obese adults were screened to follow a modified Atkins diet plan (30% of energy from protein, 40% from carbohydrate and 30% from fat). We longitudinally profiled dynamics of gut bacteria and fungi based on 16S rRNA and ITS rRNA gene sequencing data, respectively.

**Results:**

A total of 65 participants followed the modified Atkins diets for 20–231 days, with 61 and 27 participants achieving a weight loss of at least 5 and 10%, respectively. Most of the participants who achieved 10% weight loss also experienced improvements on metabolic health. The diversity of gut bacteria and fungi increased after a weight loss of 5% and kept stable thereafter. Bacteria genera including *Lachnoclostridium* and *Ruminococcus 2* from *Firmicutes* phylum were depleted, while *Parabacteroides* and *Bacteroides* from *Bacteroidetes* phylum were enriched after weight loss. The inter-kingdom analysis found an intensive covariation between gut fungi and bacteria, involving more than half of the weight loss-associated bacteria.

**Conclusions:**

This study confirmed the modulation of bacterial and fungal composition during weight loss with the low-carbohydrate diets and showed previously unknown links between intestinal bacteria and fungi accompanying the weight loss.

## Introduction

Obesity has been a worldwide public health issue, given its increasing prevalence and detrimental impact on human health (1). Previous studies comparing relative effectiveness among popular named dietary programs found that in the short term the most effective weight reduction diet was Atkins (2, 3). Atkins diet is one of the most popular low carbohydrate weight loss programs which consists of <40% of kilocalories from carbohydrates per day, ~30% from protein and 30–55% from fat (2). Facing the public health failure that obesity rates remain at historic highs despite a persistent focus on eating less and exercising more (guided by the energy balance model), recent studies are highlighting an alternative paradigm, the carbohydrate-insulin model (CIM) (4). CIM proposes that hormonal responses to a high-glycemic-load diet increases fat deposition in the body and drives positive energy balance. Thus, the Atkins diet characterized by the low carbohydrate feature may promote weight loss through the low-glycemic-load mechanism.

Accumulating evidence indicates that the gut microbiota may play a key role mediating the effect of dietary exposure on metabolic disorders such as obesity (5). For example, gut microbiota may contribute to obesity through exhibiting effects on immune system, nutrient metabolism, and hormones involved in food intake and energy harvest (6–8). A higher *Firmicutes*-to-*Bacteroidetes* ratio was observed in obese children when compared to normal weight children, and a 20% increase in the *Firmicutes* phylum abundance was associated with an increase of 150 kcal in energy harvest (7). Moreover, several studies observed that the abundance of *Firmicutes* decreased while *Bacteroidetes* increased after Rouxen-Y gastric bypass and laparoscopic sleeve gastrectomy (9–11). Human gut fungal community comprises <0.1% of the gut microbial cells. Changes in the gut fungi are linked to various diseases (e.g., inflammatory bowel disease, and colorectal cancer), indicating its potential to modulate metabolic pathways to health (12, 13). It is still unclear about the dynamic changes of gut bacteria or fungi among obese adults on a low carbohydrate (modified Atkins) diet intervention.

Therefore, in the present study, we aimed to profile the dynamics of gut bacteria and fungi over the course of diet therapy (modified Atkins diet)-induced weight loss in overweight and obese adults. Clarification of the gut microbial characteristics during the dietary intervention would help understand the potential mechanisms behind the weight loss and facilitate the discovery of new microbial targets for the obesity treatment.

## Methods

### Study Design and Participants

This study was undertaken at the Clinical Nutrition Department of the Third Hospital of Hebei Medical University. Overweight and obese adults (BMI>24.0 kg/m^2^) who came to visit the nutrition clinic between March 1, 2019 and November 30, 2020 were screened for inclusion. The inclusion criteria included 18–55 years of age and a stable body weight for at least 3 months prior to the study, defined as weight loss or weight gain <3 kg within preceding 3 months. Exclusion criteria included: concomitant use of any weight loss medication (e.g., Orlistat) or diet/exercise regime designed for weight loss; use of lipid-lowering or diabetes medications; previous bariatric, or other intestinal surgery; pregnancy or lactation; use of probiotic/prebiotic supplements; chronic use of antacids; use of antibiotics within the preceding 3 months of enrollment; clinically significant cardiovascular or respiratory diseases; liver or kidney disease; active malignancy; chronic infections; Hypothyroidism; Cushing's disease; alcohol or drug addiction; change in smoking habits within the preceding 3 months or plan to quit smoking in the following days.

After the screening and inclusion of the participants, a registered dietitian provided the enrolled participants with a structured advice for daily macronutrient, namely a uniform modified Atkins diet (total energy equals to basal metabolism, consisting of 30% total energy intake (E) from fat, 30% E from protein and 40% E from carbohydrate) for weight loss treatment. For each participant, an individualized, detailed 7-day recipe (Supplementary Table S1) were formulated by the registered dietitian. The energy intake target was designed individually according to the basic metabolic rate and was kept unchanged throughout the study. Participants were instructed to consume only the recommended foods or beverages during the following days, and were asked not to change their lifestyle and physical activity throughout the study period. All participants were required to send pictures of meals to the dietitian via WeChat app every day and then the dietitian gave timely comments and recommendations to make sure the participants complied to the required diet pattern well. At the same time, any adverse events were reported immediately via the WeChat or phone call.

Participants came back to re-visit the outpatient clinic when they achieved a weight loss of 5 and 10% from baseline. Fecal samples were collected on the day of study visit and were maintained at 4°C for no longer than 2 h. Thereafter, the samples were transported to laboratory and stored at −80°C until DNA extraction. At each visit, body weight was measured using a smart weight measuring instrument (SH-E10, Zhengzhou ShangHe Electronic Technology Co., Ltd. China), with an accuracy of 0.1 kg for the weight scale; a questionnaire on the items of the consumed foods and beverages during the past 24 h was completed for assessing compliance to the modified Atkins diet. Fasting venous blood was collected for biochemical analysis at baseline and when a weight loss of 10% was achieved. We define the period from baseline to the time point corresponding to a weight loss of 5% as the first phase of this study, while the next period lasting to the time point when 10% of the body weight were lost as the second phase. The present study focuses on one of the primary outcomes of the registered study (ClinicalTrials.gov, NCT04207879) and the other primary outcome of the registered study is still under investigation.

### Analysis of Dietary Intakes

Using the participants' records, daily nutrient intakes were determined by trained staff using the individualized evaluation software for the nutrition clinic (version 1.0, Shanghai Zhending Health Technology Co., Ltd). The individual daily intakes of macronutrients (carbohydrate, fat, and protein) and total energy were then collated for assessment of compliance to the planed Atkins diets.

### Biochemical and Clinical Chemistry Analyses

Blood pressure was examined with an automatic sphygmomanometer (CITIZEN) and body composition was measured using a bioimpedance analysis (BIA) (InBody S10; Biospace) at each visit. Clinical biochemistry analyses, including fasting glucose, glycosylated hemoglobin (HbA1c), triglycerides (TG), total cholesterol (TC), low-density lipoprotein cholesterol (LDL-C), high-density lipoprotein cholesterol (HDL-C), creatinine, uric acid (UC), aspartate transaminase (AST), and alanine transaminase (ALT) were performed with Beckman Kurt au5800 automatic biochemical analyzer (Beckman Kurt, Shanghai, China).

### Gut Microbiota Measurement and Bioinformatics Analysis

Microbial DNA was extracted from each sample using the QIAamp DNA Stool Mini Kit (Qiagen, Hilden, Germany) per the manufacturer's instruction. The final DNA concentration and purification were determined by NanoDrop 2000 UV-vis spectrophotometer (Thermo Scientific, Wilmington, USA), and DNA quality was checked by 1% agarose gel electrophoresis. The V3-V4 hypervariable region of the 16S rRNA gene was amplified with primers 338F: CCTACGGGNGGCWGCAG and 806R: GACTACHVGGGTATCTAATCC, while internal transcribed spacer 2 (ITS2) hypervariable region of the fungal ITS rRNA gene was amplified with primers ITS3F: GCATCGATGAAGAACGCAGC and ITS4R: TCCTCCGCTTATTGATATGC by thermocycler PCR system (GeneAmp 9700, ABI, USA). Purified amplicons were pooled in equimolar and paired-end sequenced (2 × 250) on an Illumina NovaSeq platform (Illumina, San Diego, USA) according to the standard protocols by Majorbio Bio-Pharm Technology Co. Ltd. (Shanghai, China). Fastq files were demultiplexed by the NovaSeq Controller Software (Illumina Inc.) using a mapping file as input. QIIME2 (version 2020.2) was used for the downstream analysis (14). The demultiplexed 16S sequences were denoised and grouped into amplicon sequence variants (ASVs; i.e., 100% exact sequence match) using DADA2 (15). The ASV features that were presented in only one sample were excluded. The taxonomies of ASVs were subsequently determined using the Naive Bayes classifier trained on the Sliva_132 99% reference database. α-diversity analysis was conducted through the q2-diversity plugin at the sampling depth of 5,000. Function prediction analysis of bacterial communities based on representative sequences was conducted using PICRUSt2 QIIME2 plugin with default settings (16). α-diversity was estimated by Shannon's diversity index (or Shannon; a quantitative measure of community richness and evenness) and Observed Features (or Richness; a qualitative measure of community richness).

For the ITS analysis, raw data was obtained using the same method as previously described. Fastq files were demultiplexed by the NovaSeq Controller Software (Illumina Inc.) and sequences were merge-paired, quality filtered and analyzed using QIIME2 (version 2020.2). As described above, we used DADA2 denoised-paired plugin in QIIME2 to process the fastq files. We filtered the features that were present in only a single sample. The individual ASVs were taxonomically classified based on the UNITE (version 8.2) database using the VSEARCH tool wrapped in QIIME2. α-diversity analysis was conducted at the sampling depth of 10,000 and estimated by the indices the same as 16S data.

### Statistical Analysis

Statistical analysis was performed using Stata version 15 and R version 3.5.3. Data with normal distribution are presented as mean (SD), those with skew distribution are presented as median (IQR). Paired *T*-test or Wilcoxon test was conducted to examine the differences in repeated measured cardiometabolic risk factors and liver and kidney function indices between study visits, including blood fasting glucose, HbA1c, triglycerides, HDL cholesterol, LDL cholesterol, total cholesterol, ALT, AST, and creatinine. Specifically, for those data with normal and skew distribution, paired *T*-test and paired Wilcoxon test were used, respectively.

We compared the alpha diversity of both gut bacteria and fungi between visits (i.e., baseline, follow-up 1 when 5% of body weight was lost, and follow-up 2 when 10% of body weight was lost) at genus level, using paired *T*-test. Principal coordinate analysis (PCoA) based on Bray-Curtis distance and permutational multivariate analysis of variance (PERMANOVA) (999 permutations) were performed to examine the changes in gut microbial community structure (β-diversity dissimilarities) along with weight loss using the *vegan* R package (v2.5-6). We applied paired Wilcoxon test on each bacterial genus or predicted pathway individually to identify specific features that changed substantially together with the decrease of body weight, and Benjamini-Hochberg method was used to correct raw *p*-values. At the same time, we used LEfSe (Linear discriminant analysis Effect Size) to calculate the LDA score of each bacterial genus between visits for supporting visualization of the results.

We separately calculated the changes (5% weight loss vs. baseline) in the relative abundance of each genus for both gut bacteria and gut fungi, and constructed alteration matrixes representing the dynamics of gut bacteria and gut fungi, respectively. We then applied the Procrustes analysis to estimate overall relationship between the two alteration matrixes. Thereafter, we examined specific correlations at genus level, by analyzing the correlations between changes (5% weight loss vs. baseline) in the relative abundance of each genus stratified by the kingdom, using Spearman correlation analyses.

## Results

### Characteristics of the Participants

**Figure 1:**
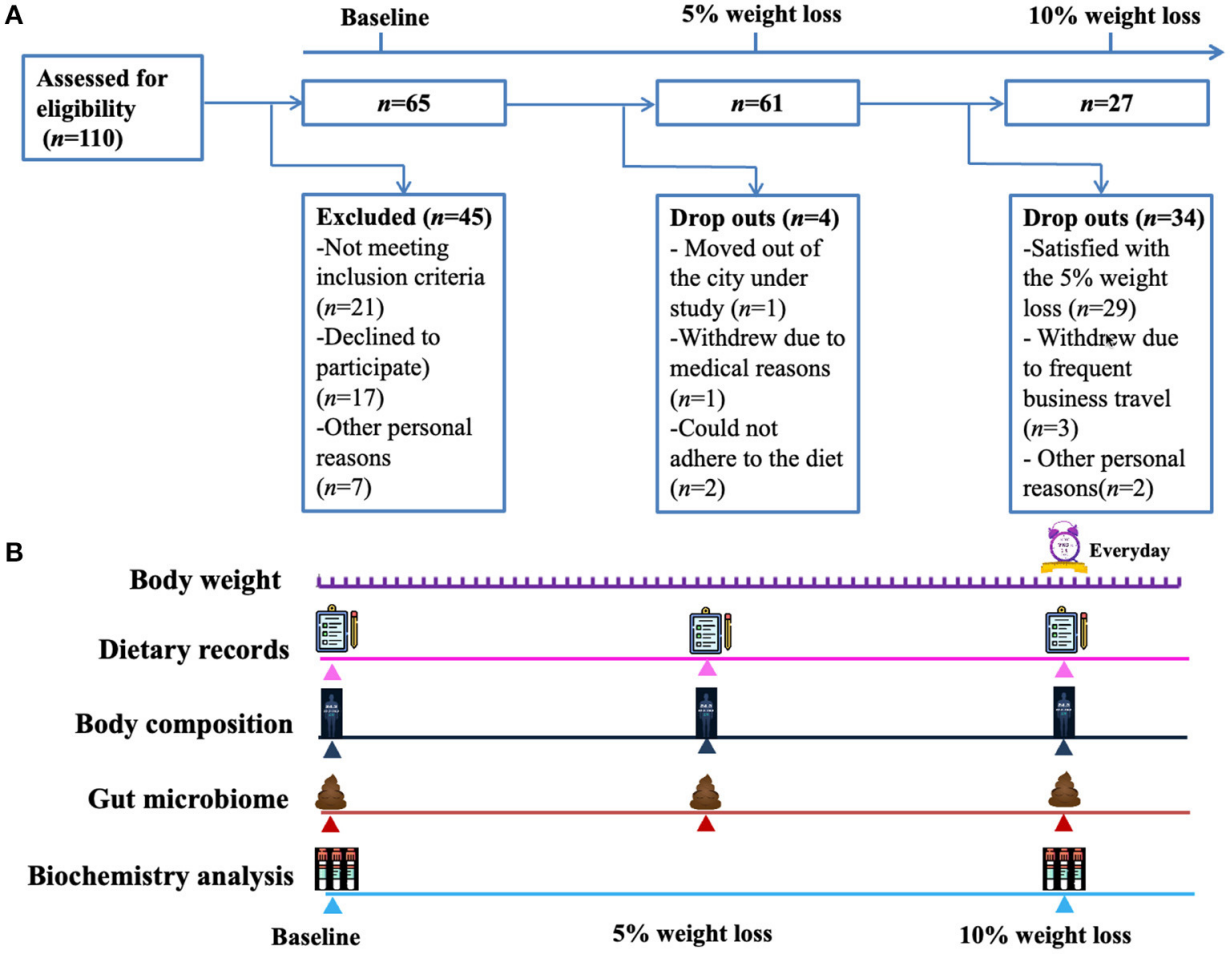
Study flow chart and design. **(A)** A total of 110 subjects were screened and 65 subjects were included, of which 4 dropped out before achieving 5% reduction of body weight, remaining 61 participants achieving a weight loss of at least 5% and 27 participants achieving a weight loss of at least 10%. **(B)** The included participants reported their body weight to investigators every day, and visited the outpatient clinic when they achieved a weight loss of 5 and 10% from baseline. At each visit, a questionnaire on the items of the consumed foods and beverages during the past 24 h was completed to assess the compliance to the modified Atkins diet. Fasting venous blood samples were collected for biochemical analysis at baseline and when a weight loss of 10% was achieved.

**Figure 2:**
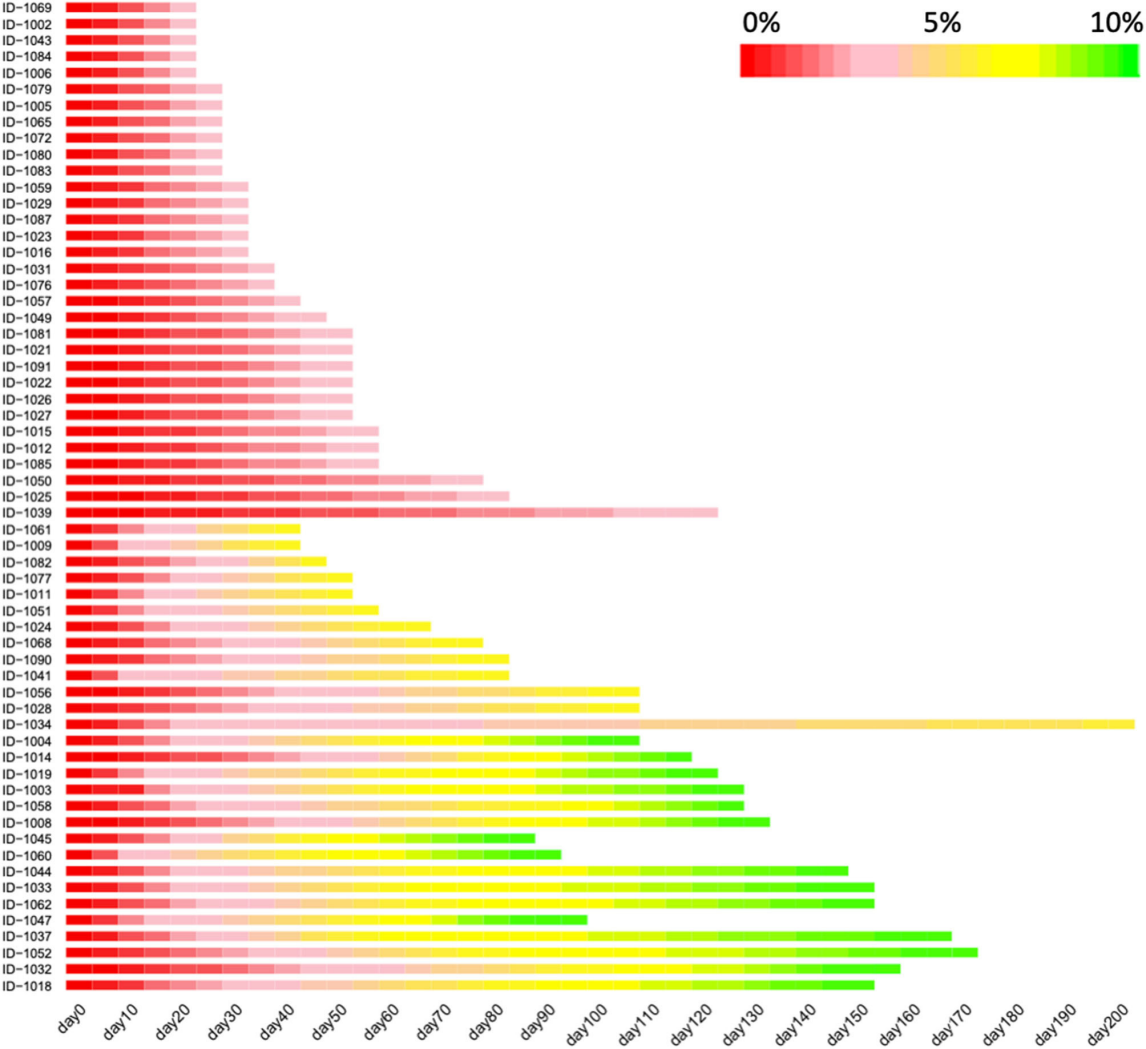
The velocity of body weight reduction over the course of low-carbohydrate diet program. Heatmap depicting the values of the percentage of lost body weight, with red color indicating the baseline body weight while yellow and green indicating that the percentage reach 5 and 10%, respectively. Only the participants achieving a weight loss of at least 5% were included in this figure (*n* = 61). This figure was trimmed at 150 days due to the space limits.

A total of 65 subjects (male, *n* = 20; female, *n* = 45) were enrolled, and the average baseline characteristics of the participants were: age 36 ± 9 years (range 19–55), BMI 30.1 ± 4.5 kg/m^2^ (range 24.2–48.5), body weight 83.0 ± 16.0 kg (range 60.0–142.1), and basic metabolic rate (BMR) 5.7 ± 0.8 MJ/day (range 4.6–7.7). The detailed characteristics were summarized in the Table 1. Of the 65 participants, 4 dropped out before achieving 5% reduction of body weight, remaining 61 participants achieving a weight loss of at least 5% and 27 participants achieving a weight loss of at least 10% (Figure 1). For the 61 participants achieving at least 5% reduction of body weight from baseline, the interval ranged from 7 to 196 days, with a median of 25 days. For the 27 participants who attended the third visit (i.e., 10% reduction of body weight was achieved), the median interval between the second and third visit was 46 days (ranging from 21 to 231 days). Heatmap plot indicating the mean velocity of body weight reduction between visits are shown in the Figure 2.

**Table 1:** Baseline demographics of the study participants.

**Items**	**Values**
**Sex, no. (%)**
Male	20 (31)
Female	45 (69)
**Age, mean (SD), y**	36.0 (9.0)
**Highest level of education achieved, no. (%)**
High school graduate	18 (28)
College graduate	30 (46)
Post-graduate degree	17 (26)
**Weight, mean (SD), kg**
Male	93.5 (9.3)
Female	79.3 (16.3)
Both	83.0 (16.0)
**Body mass index, mean (SD), kg/m** ^**2**^
Male	30.8 (2.8)
Female	29.8 (5.0)
Both	30.1 (4.5)
**Basic metabolic rate, mean (SD), MJ**
Male	6.7 (0.4)
Female	5.3 (0.5)
Both	5.7 (0.8)
**Overweight/obese**
Overweight (%)	22 (33.8%)
Obese (%)	43 (66.2)

### Compliance to Dietitian-Designed Low-Carbohydrate Diet

At baseline, data from food records demonstrated that about 52.2% of the participants' daily energy intake were from carbohydrates, 33.8% from fat, and 14.0% from protein (Supplementary Figure S1A). Following the dietitians' recommendation, the percentage of energy were 32.0, 42.2, and 25.8% for carbohydrate, fat and protein, respectively, during the phase 1 period of this study (Supplementary Figure S1B). Similar distribution of the macronutrients continued to the third visit for those who achieved a weight loss of 10%, with mean percentages of 32.8, 41.8, and 25.4% for carbohydrate, fat, and protein, respectively (Supplementary Figure S1C). These data suggested that the participants complied well to the dietitian-designed Atkins diet recommendations.

### Body Composition and Cardiometabolic Risk Factors

**Figure 3:**
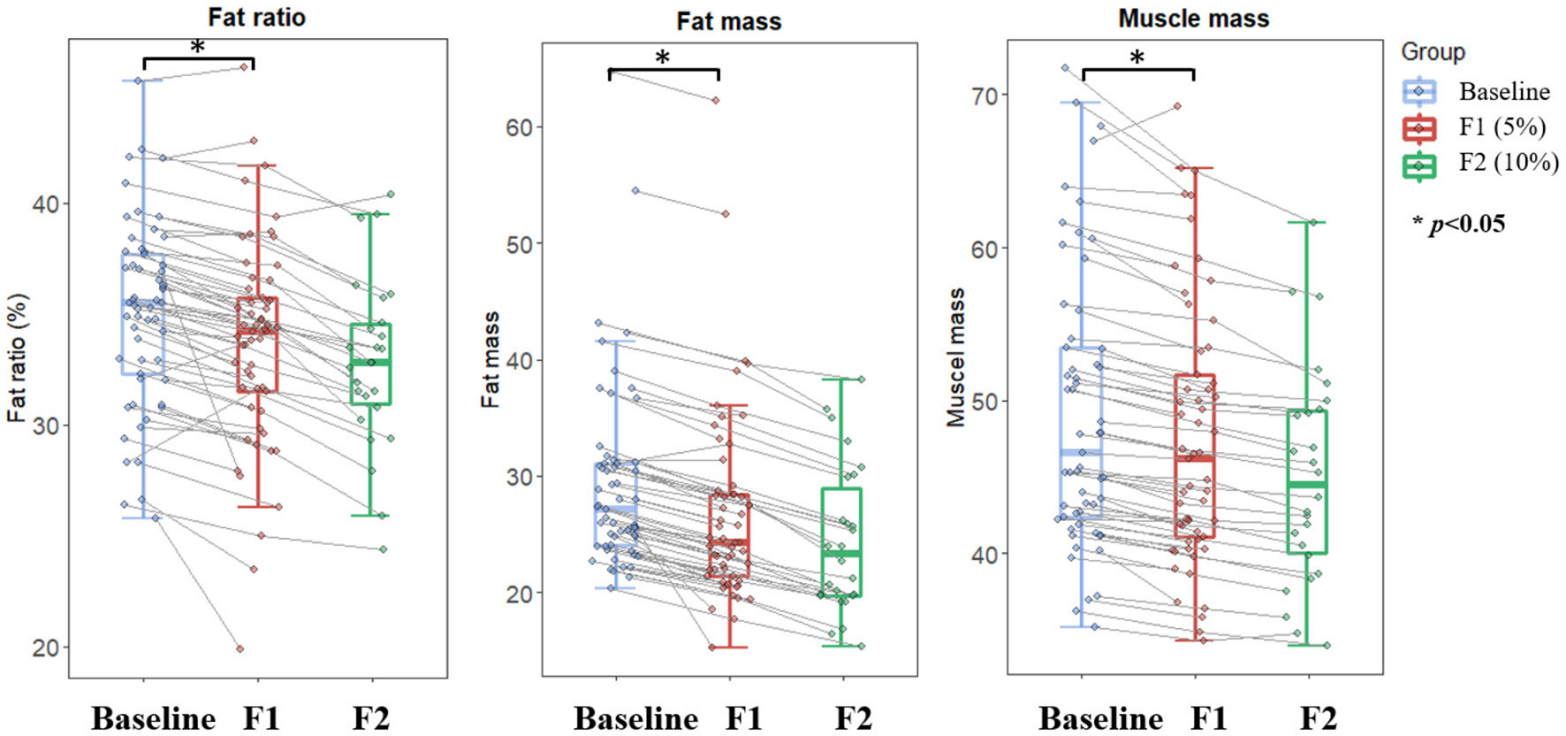
Changes in body composition over the course of low-carbohydrate diet-induced body weight. Boxplot depicting fat ratio, fat mass, and muscle mass at different timepoints corresponding to baseline (light blue color), 5% weight loss (red color), and 10% weight loss (green color), respectively. Lines between boxplots link body composition values of the same participant at different timepoints. Paired *T*-test was conducted to examine difference between sequential visits, with *p < 0.05 indicating statistical significance.

The body composition analysis demonstrated that both body fat and body muscle significantly decreased along with the body weight loss (Figure 3). Specifically, the body fat decreased by about 8.7 and 18.6% at the first and the second visit from baseline, respectively. Meanwhile, body muscle decreased by 3.2 and 9.1%, respectively. Accordingly, the body fat percentage continued decreased from 35.1 to 33.7% at the end of phase 1, and further to 32.5% at the end of phase 2.

**Figure 4:**
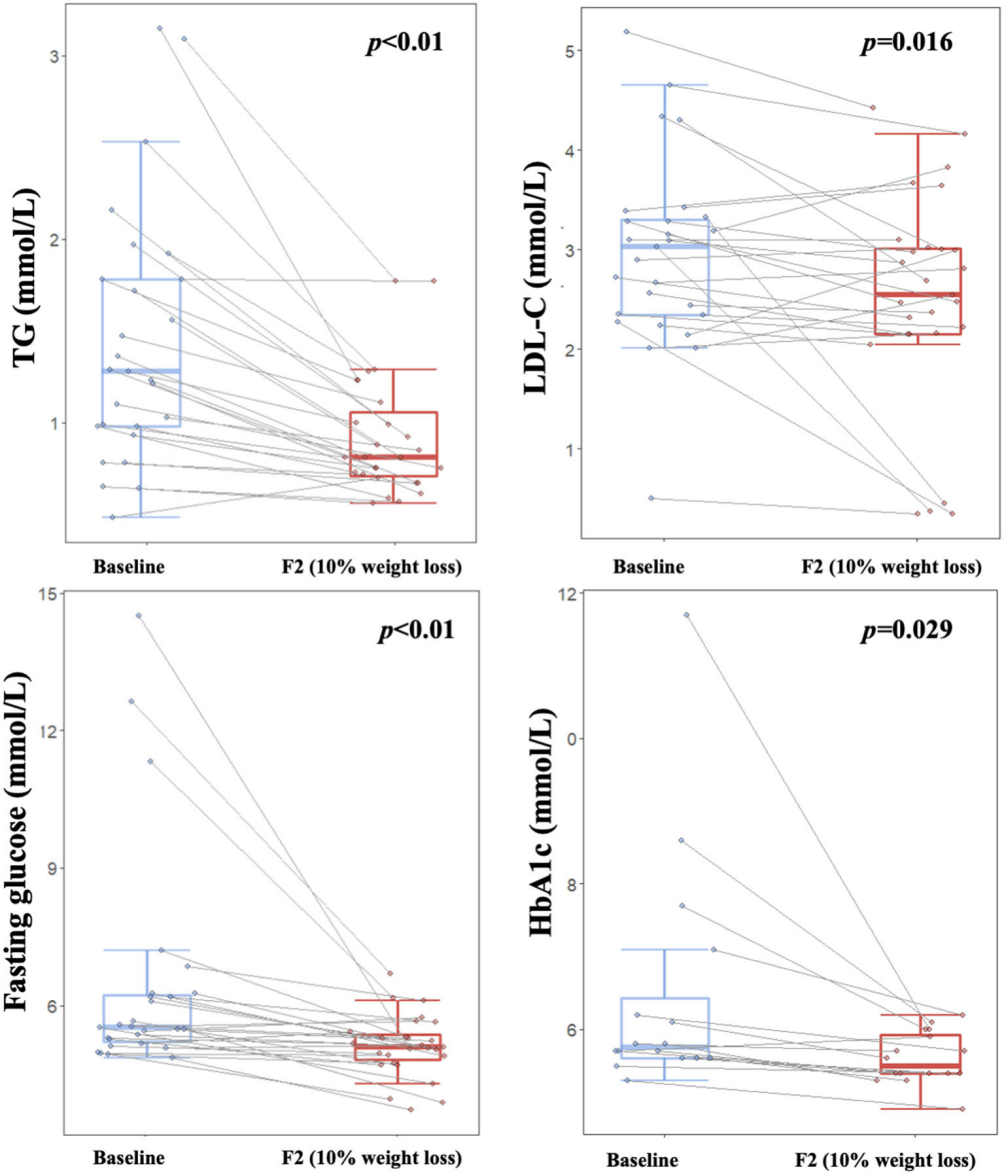
Improvements on lipid metabolism and glucose homeostasis over the course of low-carbohydrate diet-induced weight loss. Boxplot depicting TG, LDL, blood glucose, and HbA1c levels at different timepoints corresponding to baseline (light blue color) and 10% weight loss (red color), respectively. Lines between boxplots link values of the same participant at different timepoints. Paired *T*-test was applied to examine difference between visits, with *p* < 0.05 indicating statistical significance. TG, Triglycerides; LDL-C, Low-density lipoprotein cholesterol; HbA1c, Glycosylated hemoglobin.

Our study showed that blood lipid profile and glucose homeostasis were improved during the dietary intervention. Blood TG and LDL-C were decreased significantly after a weight loss of 10% (*p* < 0.05, Figure 4), while no significant changes were found for TC or HDL-C (Table 2). Both fasting glucose and HbA1c were decreased significantly (*p* < 0.05, Figure 4), especially for those with relatively higher baseline values. Additionally, both ALT and AST were significantly decreased (*p* < 0.01, Table 2), which indicated a short-term safety of the low-carbohydrate diets. Furthermore, no adverse events were reported during the study.

**Table 2:** Macronutrient distribution, metabolic health, and gut microbial features at each visit accompanying weight loss.

	**Baseline**	**5% Weight loss**	***P*-value**	**10% Weight loss**	***P*-value**
	**(*n =* 61^#^)**	**(*n =* 61)**	**(F1 vs. BL)**	**(*n =* 27)**	**(F2 vs. BL)**
**Dietary intakes**
Total energy intake (kcal)	1805.3 (1303.6–2254.6)	996.5 (797.9–1212.4)	<0.001	1091.3 (934.7–1320.0)	<0.001
Carbohydrates (g)	233.6 (151.9–315.1)	76.7 (55.8–113.5)	<0.001	94.4 (55.9–124.4)	<0.001
Fat (g)	63.8 (51.1–85.5)	45.6 (37.9–57.9)	<0.001	50.0 (41.2–61.9)	<0.001
Protein (g)	64.2 (41.7–77.7)	65.6 (51.1–78.5)	0.733	72.9 (57.0–84.6)	0.186
Fiber (g)	12.7 (8.9–16.9)	11.8 (8.4–15.9)	0.738	13.6 (11.0–18.4)	0.683
**Body composition**
BMI (kg/m^2^)	30.2 (4.5)	28.6 (4.4)	<0.001	26.73 (3.5)	<0.001
Fat ratio (%)	35.1 (3.7)	33.7 (3.5)	<0.001	32.8 (3.8)	<0.001
Fat mass (kg)	29.3 (8.1)	26.8 (8.2)	<0.001	23.9 (6.4)	<0.001
Muscle mass (kg)	49.2 (9.0)	47.6 (8.6)	<0.001	44.7 (7.0)	<0.001
**Blood pressure**
Systolic (mmHg)	126.6 (3.5)	121.9 (4.5)	0.028	115.8 (2.9)	0.001
Diastolic (mmHg)	89.2 (2.5)	83.4 (3.7)	0.002	83.3 (2.6)	0.002
**Blood lipids**
Triglycerides (mmol/L)	1.4 (0.1)	NA	NA	0.9 (0.1)	<0.001
Total cholesterol (mmol/L)	4.9 (0.2)	NA	NA	4.6 (0.2)	0.055
HDL–cholesterol (mmol/L)	1.3 (0.1)	NA	NA	1.3 (0.1)	0.953
LDL- cholesterol (mmol/L)	3.0 (0.2)	NA	NA	2.5 (0.2)	0.016
**Glucose homeostasis**
HbA1c (%)	6.5 (0.4)	NA	NA	5.6 (0.9)	0.029
Fasting glucose (mmol/L)	6.4 (0.5)	NA	NA	5.1 (0.1)	0.004
**Liver and kidney function indices**
AST (U/L)	23.1 (2.5)	NA	NA	16.5 (0.8)	0.008
ALT (U/L)	37.2 (6.3)	NA	NA	18.3 (1.7)	0.002
Creatinine (μmol/L)	60.5 (2.7)	NA	NA	62.8 (2.4)	0.124
Uric acid (μmol/L)	369.8 (20.4)	NA	NA	356.3 (17.4)	0.301
Gut microbial characteristics^*^
Shannon (bacteria)	5.2 (4.65–5.59)	5.3 (4.9–5.9)	0.031	5.3 (4.8–5.7)	0.221
Observed features (bacteria)	159.0 (112.0–181.0)	148.5 (120.0–190.0)	0.034	142.0 (116.0–171.0)	0.330
*Firmicutes*–to-*Bacteroidetes* (%)	20.2 (6.36–40.89)	6.4 (2.82–21.03)	0.002	7.3 (4.3–14.3)	0.701
Shannon (fungi)	2.4 (1.0–3.0)	3.1 (2.2–3.5)	0.009	3.2 (2.5–3.5)	0.016
Observed features (fungi)	37.0 (23.0–50.0)	38.0 (26.0–56.0)	0.113	34.0 (25.0–54.0)	0.631

*Data with normal distribution are presented as mean (SD), those with skew distribution are presented as median (IQR). For those data with normal and skew distribution, paired T-test and paired Wilcoxon test were used to assess significance of the difference, respectively. BL, baseline*.

# *65 participants were included in the present study, with data of 61 participants available for the paired analysis*.

* *n = 60 for the comparison between baseline and F1, as 1 participant did not provide stool sample at F1 visit*.

### Dynamic Changes of Gut Bacteria in the Course of Modified Atkins Diet-Induced Weight Loss

**Figure 5:**
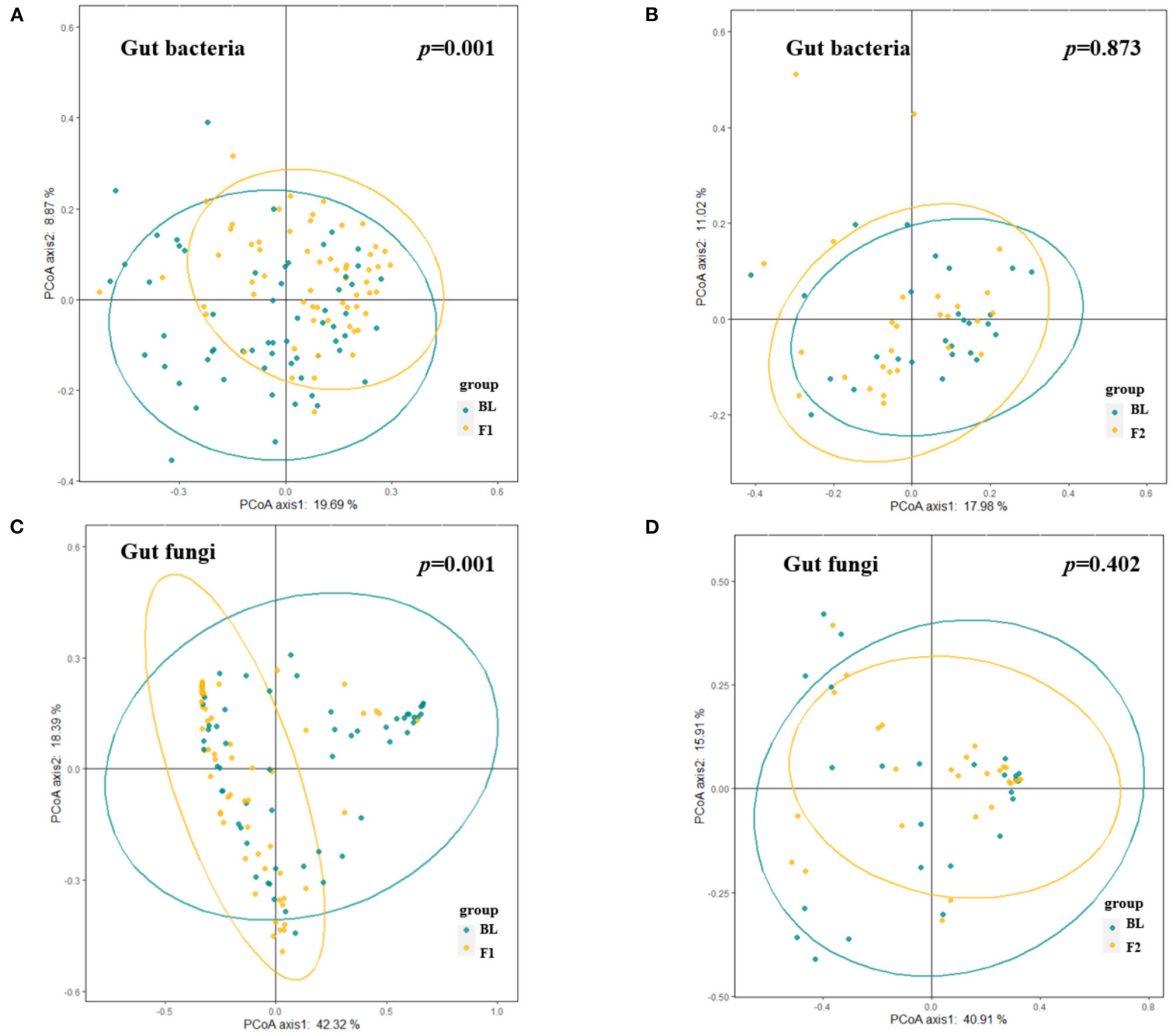
Alteration in gut microbial composition over the course of low-carbohydrate diet-induced weight loss. **(A,B)** Dissimilarities in gut bacterial composition at different visits [**(A)**, baseline vs. 5% weight loss; **(B)**, 5% weight loss vs. 10% weight loss] represented by unconstrained principal coordinate analysis (PCoA) with the Bray Curtis dissimilarity index. **(C,D)** Dissimilarities in gut fungal composition at different visits [**(C)**, baseline vs. 5% weight loss; **(D)**, 5% weight loss vs. 10% weight loss] represented by unconstrained PCoA with the Bray Curtis dissimilarity index. PERMANOVA *p* < 0.05 indicates significant explanation of weight loss for the dissimilarities in gut microbiota composition.

**Figure 6:**
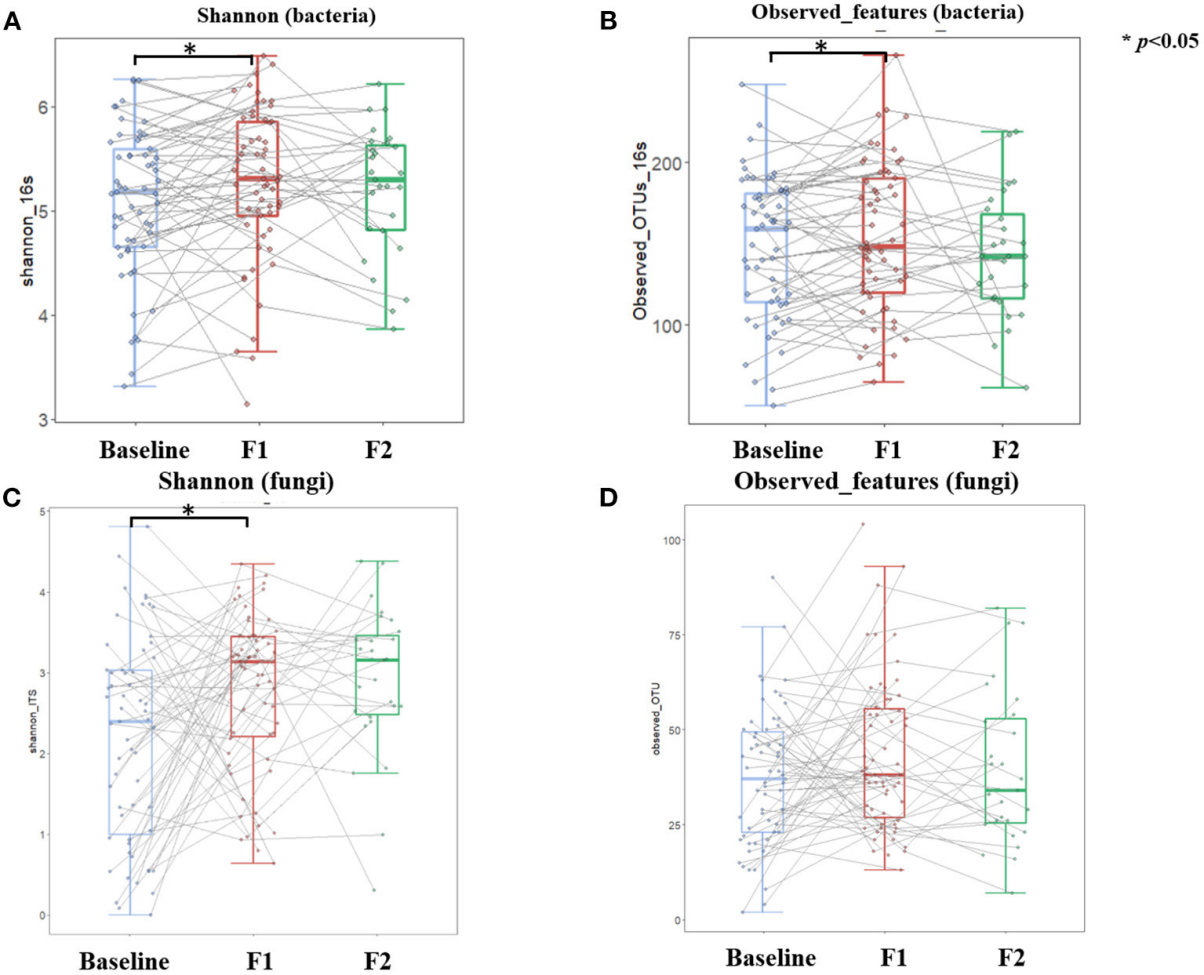
Dynamics of gut microbial diversity over the course of low-carbohydrate diet-induced weight loss. Boxplot depicting Shannon and Observed features of both gut bacteria **(A,B)** and gut fungi **(C,D)** at different timepoints corresponding to baseline (light blue color), 5% weight loss (red color), and 10% weight loss (green color), respectively. Lines between boxplots link body composition values of the same participant at different timepoints. Paired *T*-test was conducted to examine difference between sequential visits, with ^*^p < 0.05 indicating statistical significance.

A total of 120 gut bacteria genera were detected in more than 90% of the samples, and these genera mainly belonged to the phylum of *Firmicutes, Bacteroidetes, Actinobacteria*, and *Proteobacteria*. At the phylum level, we investigated dynamics of the bacteria distributions and found that the relative abundance of *Bacteroidetes* increased while *Firmicutes* and *Proteobacteria* decreased after a weight loss of 5%. This dynamic also reflected the decreased trend of *Firmicutes*-to-*Bacteroidetes* ratio, along with the body weight loss in the phase 1 stage. However, this alteration trend was reversed to some extent after a weight loss of 10% (Supplementary Figure S2). Figure 5 showed the dissimilarities (β diversity) in gut bacterial composition of samples collected at different time points, which were represented by unconstrained principal coordinate analysis (PCoA). Unlike the body fat percentage which decreased at a relatively constant velocity along with the weight loss, the gut bacteria composition experienced dramatic alteration during the first phase of weight loss (Figure 5A, PEMANOVA test, *p* = 0.001), while keeping relatively stable during the second phase (Figure 5B, PEMANOVA test, *p* = 0.87). Of note, half of the enrolled participants did not achieve a weight loss of 10%, and thus were not included in the phase 2 analysis. Regarding the α-diversity of gut bacteria, Figure 6 showed similar trends that both genus-level Shannon index and observed features significantly increased as the body weight decreased during the phase 1 stage (*p* < 0.05). But no significant changes in these indices were observed during the second phase.

We further identified which microbes changed substantially during each stage at genus level and found that most of the depleted genera including *Ruminococcus 2, Lachnoclostridium, Parasutterella, Escherichia-Shigella, Klebsiella, Parasutterella* were taxonomically belonging to *Firmicutes* or *Proteobacteria* phylum (Supplementary Figure S3). Of note, only *Ruminococcus 2* and *Lachnoclostridium* could be validated at both timepoints corresponding to a weight loss of 5 and 10%, respectively. On the other hand, *Parabacteroides* and *Bacteroides* belonging to *Bacteroidetes* were enriched after a weight loss of 5 and 10% (Supplementary Figure S3). No significantly altered genera were identified between the end of phase 1 and the end of phase 2, which consistent with the dynamic changes of α diversity and β diversity. We also predicted the function of gut bacteria, and found 38 significantly altered pathways at the end of phase 1. More than half of the altered pathways were related to biosynthesis or degradation of amino acids, organic acids, glucose and heme (Supplementary Figure S4). The relative abundance and composition of the gut bacteria functional pathways kept stable during the second phase, and no significantly altered pathways were observed.

### Dynamic Changes of Gut Fungi in the Course of Modified Atkins Diet-Induced Weight Loss

There was a total of 99 gut fungal genera where *Ascomycota, Basidiomycota*, and unclassified *Fungi sp*. were the most dominant phyla with a mean relative abundance of 42.4, 7.6, and 49.9%, respectively. The relative abundance of *Ascomycota* decreased while the *Basidiomycota* increased after a weight loss of 5% at the end of phase 1, and this trend continued in 27 participants who further achieved a weight loss of 10% at the end of phase 2 (Supplementary Figure S2). After 10% prevalence filtering, 33 gut fungal genera were identified as a group of core taxa, including *Saccharomyces, Candida, Aspergillus, Malassezia*, etc. Compared with the baseline measures, the α-diversity analysis showed that Shannon index increased significantly after a weight loss of 5% at the end of phase 1 (Figure 6C). Thereafter, the Shannon index was relatively stable with no significant changes observed during the second phase (Figure 6C). Unlike the gut bacteria that showed an increase in observed features along with the weight loss, this index of α-diversity (i.e., observed features) was stable for gut fungi throughout the study (Figure 6D), indicating that the gut fungal richness is less responsive to diet changes or weight loss. β diversity analysis yielded similar results, which showed dramatic alteration of gut fugal composition during the first phase of weight loss (Figure 5C, PEMANOVA test, *p* = 0.001), while keeping relatively stable during the second phase (Figure 5D, PEMANOVA test, *p* = 0.402).

### Co-variation Between Gut Bacteria and Fungi During the Atkins Diet-Induced Weight Loss

**Figure 7:**
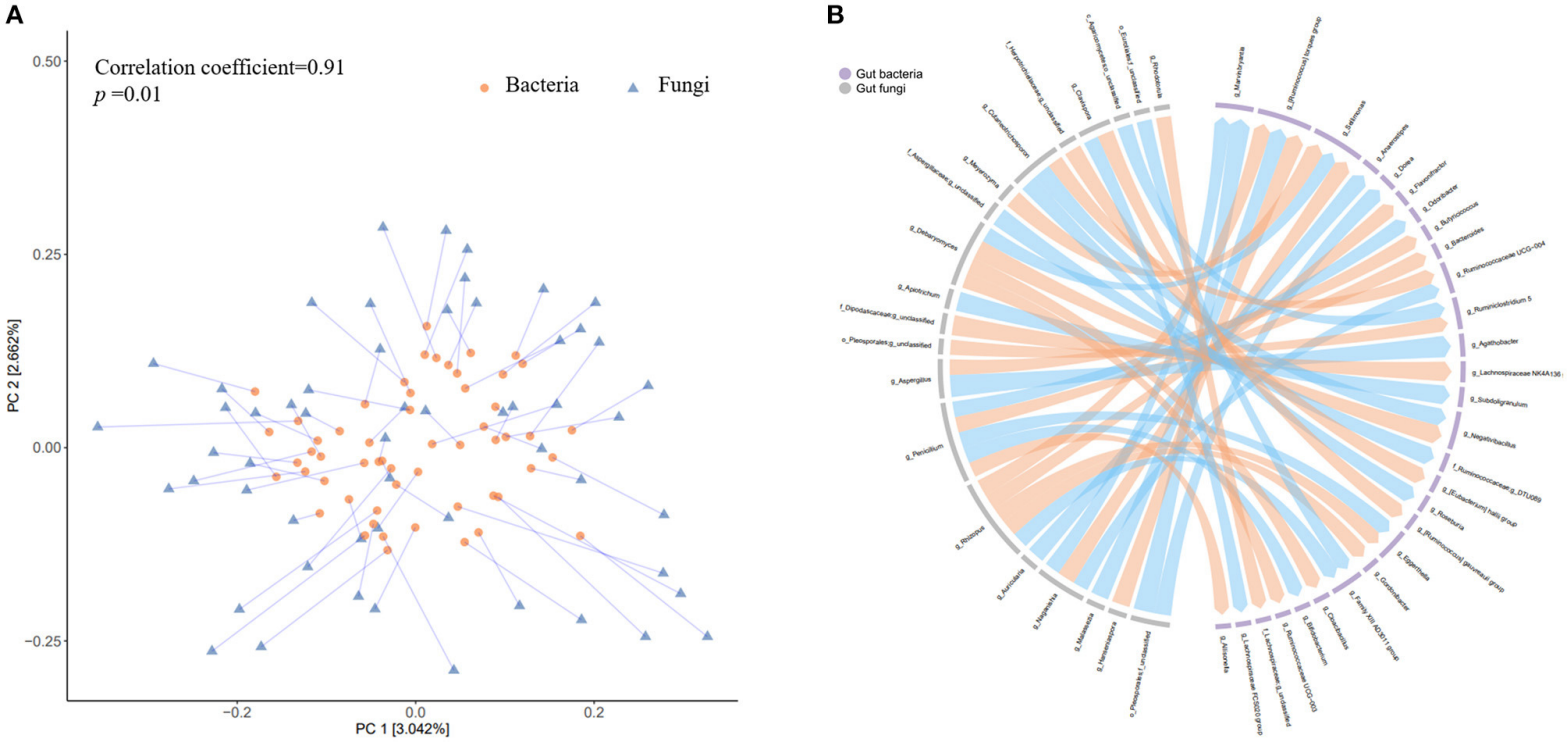
Co-variation between the changes in gut bacteria and fungi abundance accompanying weight loss. **(A)** The circles represent projections of the alteration matrix (5% weight loss vs. baseline) of gut bacterial composition and the triangles represent projections of the alteration matrix (5% weight loss vs. baseline) of gut fungal composition. Each line links projections representing the same participant, with the length indicating distance. **(B)** The left part of the circle colored in gray denotes gut fungi at genus level, while the right part in light purple indicates gut bacteria at genus level. The links indicate there are nominally significant associations (Spearman correlation analyses, *p* < 0.01) between changes in the relative abundance (5% weight loss vs. baseline) of the two kingdoms, with orange color representing positive associations and blue color representing negative associations.

To further explore the potential co-variation between the gut fungi and bacteria, we firstly calculated the changes in relative abundance of each fungus and bacteria. The Procrustes analysis revealed a closed overall relationship between the dynamic changes of the two kingdoms (*p* = 0.01, Figure 7A). A total of 42 significant associations were identified between 20 fungi and 29 bacteria (*p* < 0.01, Figure 7B) The fungal genus *Penicillium* and another two unclassified genera belonging to family *Dipodascaceae* and order *Pleosporales* were significantly associated with 3 of the 5 bacterial genera (i.e., *Bacteroides, Ruminiclostridium 5*, and *Lachnospiraceae NK4A136 group*) which were consistently validated to be enriched after weight loss.

These results suggested extensive co-variation associations between gut fungi and bacteria, including those might be involved in the process of weight loss. Additionally, regarding the gut bacterial dynamics, most of the identified associations (29/42) were related to genera from *Lachnospiraceae* and *Ruminococcaceae* family. For the gut fungi genera that showed concordant dynamics with bacteria were mainly from *Debaryomycetaceae, Trichosporonaceae, Rhizopodaceae*, and *Aspergillaceae* family, while those showed opposite dynamics with bacteria were mainly from *Filobasidiaceae* and *Aspergillaceae* family. The positive correlation of the fungi dynamics (e.g., *g_ Debaryomyces, g_ Penicillium*, and *g_ Cutaneotrichosporon*) with bacteria dynamics (e.g., *g_Eggerthella, g_Negativibacillus, g_Selllimonas*) suggested that there might be a co-dependence for both kingdoms during the low-carbohydrate diet-induced weight loss. The inverse correlation of other fungi (e.g., *g_ Naganishia* and *g_ Aspergillus*) with bacteria (e.g., *g_Marvinbryantia and g__Ruminococcus torques group*) indicated that there might be competitive or inhibitory actions. These results in together suggest that the gut fungi have potential to manipulate the neighboring bacterial community or vice versa over the course of low-carbohydrate diet-induced weigh loss.

## Discussion

Our present study confirmed the effectiveness of the modified Atkins diets, one of the typical low-carbohydrate diets, on body weight management and metabolic health among obese adults. We profiled the characteristics and dynamics of gut bacterial and fungal features based on longitudinal data during the Atkins diet-induced weight loss. We further identified several key gut microbes which were altered during the intervention. We provide clues of close connection and covariation between gut fungi and bacteria in the process of weight loss. Our findings might extend our insight into the potential of gut fungi to indirectly impact host metabolism by influencing the numerous bacteria.

In this study the modified-Atkins diet effectively improved the obesity-associated phenotypes, including body weight, blood lipids profile, and blood glucose homeostasis. Of note, the time spent for achieving a weight loss of 5% are largely varied among the participants, and the potential characteristics that may determine the velocity of weight loss warrant further investigations. A recent study found that participants with a higher relative abundance of genus *Bacteroides* at baseline exhibited a better response to low-carbohydrate diet intervention and achieved greater weight loss outcomes (17). Interestingly, we also tried to replicate this association to some extent in the present study, but we did not find significant difference in the abundance of baseline *Bacteroides* between participants whose body weight responded quickly to the low-carbohydrate diet and those with a low velocity of weight loss. The different study designs (e.g., application of moderate calorie restriction) of the two studies may help explain the inconsistency. Nevertheless, we did observe that the abundance of genus *Bacteroides* increased significantly at both visits after 5 and 10% of the body weight were lost on the low-carbohydrate diets.

We found that the microbial composition was improved toward a lean microbiome phenotype, showing a higher alpha-diversity and *Firmicutes*-to-*Bacteroidetes* ratio. These results are consistent with those of previous studies that the ratio of *Firmicutes*-to-*Bacteroidetes* was higher in obese individuals than lean individuals, and this ratio was decreased with weight loss (8). At genus level, both of the depleted genera that were confirmed after 5 and 10% weight loss in the present study belong to the *Firmicutes* phylum, while serval of the enriched genera after weight loss were assigned to the *Bacteroidetes* phylum. Although some of the identified specific genera such as *Bacteroides* and *Parabacteroides* have been reported to be significantly increased after low-carbohydrate diet-induced weight loss, the alterations were not specific to the low-carbohydrate diet (18). Interestingly, the above-mentioned alterations in gut bacterial composition only appeared when compared with baseline samples, while no significant alterations were identified between 5% weight loss and 10% weight loss. This dynamic pattern also applied to the gut bacteria functional analysis. These findings are to some extent consistent with a recent study which reported resilience of the gut microbiota after a long-term dietary intervention (18).

A diverse community of fungi coexists in the gut with other members of the microbiota (19–21). To the best of our knowledge, this is the first study that profiled the fungal dynamic changes and their covariations with gut bacteria over the course of low-carbohydrate diet-induced weight loss among obese participants.

Our study found that the gut fungi were dominated by members of the phylum *Ascomycota*, which included the genera *Candida* and *Saccharomyces*. The ecology of fungal and bacterial cohabitation in the gut has great potential to modulate metabolic health. Diet and nutrition might play a key role in shaping this ecosystem (22–24). Our study did observe alterations in this ecosystem over the course of the low-carbohydrate diets-induced weight loss. We also observed some differences in the alterations between fungi and bacteria. For example, both evenness and richness of gut bacteria increased with the introduction of low-carbohydrate diets, which might be explained by the needs of more diverse bacteria for fermenting some newly introduced food components. As a contrast, gut fungi experienced increases mainly in evenness but not richness throughout the study, indicating the stability of the harbored fungal taxa which may not be dependent on the diet-derived components or nutrients.

Recent findings suggested a unique role for gut fungi beyond their direct effect on the host, but through their cross-kingdom interaction with gut bacteria (22). The modes of cross-kingdom interaction can be expected to include mutualism, commensalism, amensalism, parasitism and competition (20). These cross-kingdom interactions may be main factors that determine the fungi richness. Moreover, our cross-kingdom analysis provided some clues for the mutualism or competition between gut fungi and bacteria over the course of low-carbohydrate diet induced weight loss. Extensive co-variation associations were identified, with most of the associations related to bacterial *Lachnospiraceae* and *Ruminococcaceae* family, covering 3 of the 5 consistently confirmed enriched bacteria genera (e.g., *Ruminococcaceae UCG-013* and *Ruminiclostridium 5*) after weight loss. These findings implied that the fungi inhabiting the intestinal tract had the potential to influence the neighboring bacteria, and thus possibly indirectly influencing the host metabolism (22).

This study has several strengths. First, this study longitudinally collects detailed phenotype data and biological samples with an interval of fixed weight loss of 5%. This study design makes the intra-individual paired analysis more precisely reflect the weight-loss related dynamic changes. Second, this study investigates dynamic changes of gut bacteria and fungi simultaneously, and provided novel clues of the close links between intestinal fungi and bacteria over the course of weight loss, which is rarely achieved in prior studies.

A major limitation of the present study is that the number of participants reaching a 10% weight loss is relatively small, which limits the power of the statistical analysis for the second phase of the study. Another limitation is that the present analysis with an observational nature aims to profile the dynamics of gut microbial features accompanying low-carbohydrate diet induced weight loss, which is not capable to illustrate the causal relationship between gut microbial alterations and weight loss. Nevertheless, the profiling of gut microbial features, especially the close links and covariation between gut bacteria and fungi in the process of weight loss, may provide potential targets of intervention for body weight management and highlight that the role of gut fungi warrants more investigations.

## Conclusion

In conclusion, we confirmed the modulation of bacterial and fungal composition during weight loss with the low-carbohydrate diet, and profiled dynamics and characteristics of the gut bacterial and fungal cohabitation ecosystem. The identified previously unknown links between intestinal fungi and bacteria over the course of weight loss highlight that gut fungi may be much more important than expected in the development or treatment of obesity. A complete understanding of this cross-kingdom interaction on body weight and developments of microbiota-targeting diets for weight loss require further investigations.

## Data Availability Statement

The datasets presented in this study can be found in online repositories. The names of the repository/repositories and accession number(s) can be found at: https://omics.lab.westlake.edu.cn:7001/, publicly accessible.

## Ethics Statement

The studies involving human participants were reviewed and approved by Medical Ethics Committee of the Third Hospital of Hebei Medical University. The patients/participants provided their written informed consent to participate in this study.

## Author Contributions

HD and YF designed and supervised the study. DY, LX, WC, JQ, JZ, ML, YW, HT, XL, ZM, CX, SX, MS, and JL were involved in the recruitment of participants and the acquisition of data. YF and DY performed the statistical analyses, interpreted the data, and drafted the manuscript. HD revised the article critically for important intellectual content. All authors approved the final version of the manuscript to be submitted.

## Funding

This study was funded by the National Natural Science Foundation of China (82103826), Medical Science Research Project of Hebei Provincial Health Commission (Key Science and Technology Research Program, No.20210299), and Zhejiang Provincial Natural Science Foundation of China (LQ21H260002). The funders had no role in study design, data collection and analysis, decision to publish, or writing of the manuscript.

## Conflict of Interest

The authors declare that the research was conducted in the absence of any commercial or financial relationships that could be construed as a potential conflict of interest.

## Publisher's Note

All claims expressed in this article are solely those of the authors and do not necessarily represent those of their affiliated organizations, or those of the publisher, the editors and the reviewers. Any product that may be evaluated in this article, or claim that may be made by its manufacturer, is not guaranteed or endorsed by the publisher.
