# Supplementary material for: Dynamics of the Gut Bacteria and Fungi Accompanying Low-Carbohydrate Diet-Induced Weight Loss in Overweight and Obese Adults
Source: Front Nutr. 2022 Feb 11;9:846378. doi: 10.3389/fnut.2022.846378 (PMC8873986; doi:10.3389/fnut.2022.846378)
Supplement: Supplementary file 1 [file Data_Sheet_1.docx]

**Supplemental files**

**Dynamics of the gut bacteria and fungi accompanying low-carbohydrate diet-induced weight loss in overweight and obese adults**

Dan Yu^1^, Libin Xie^2^, Wei Chen^3^, Jin Qin^4,5^, Jingjing Zhang^6^, Min Lei^1^, Yue Wang^1^, Hongge Tang^1^, Xinxiu Liang^7^, Zelei Miao^7^, Congmei Xiao^7^, Sujuan Xue^1^, Meishuang Shang^4,5^, Jie Lu^6^, Hailing Di^1^*, Yuanqing Fu^7,8,9^*

**Supplemental Figures**

**Figure S1 Macronutrient distribution at each visit accompanying weight loss**. The relative abundance of bacteria (**A**) baseline, (**B**) 5% weight loss (F1), (**C**) 10% weight loss (F2). CHO, carbohydrate.

**
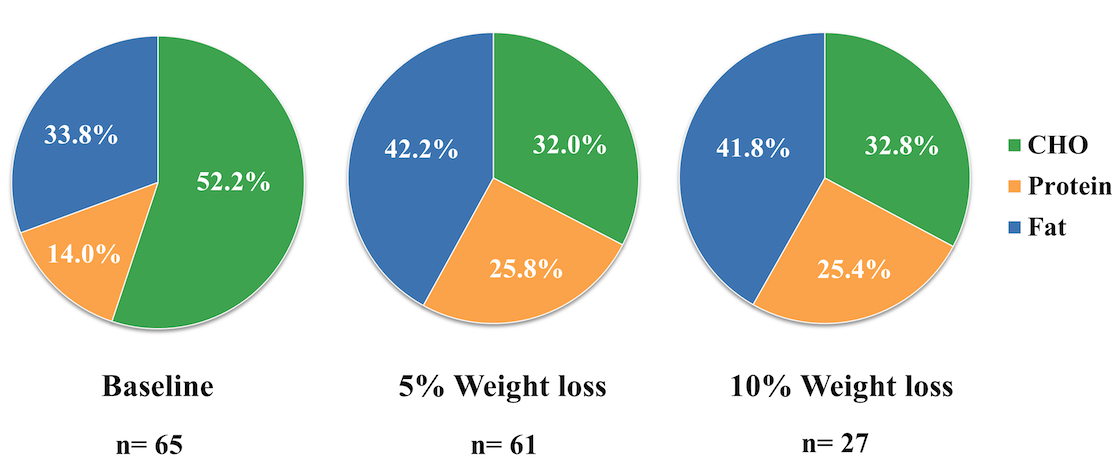
**

**Figure S2 Dynamics of gut microbiota distribution at phylum level over the course of low-carbohydrate diet-induced weight loss.** The relative abundance of bacteria (**A**) and fungal (**B**) phyla among participants at different visits corresponding to baseline, 5% weight loss (F1) and 10% weight loss (F2), respectively.

**
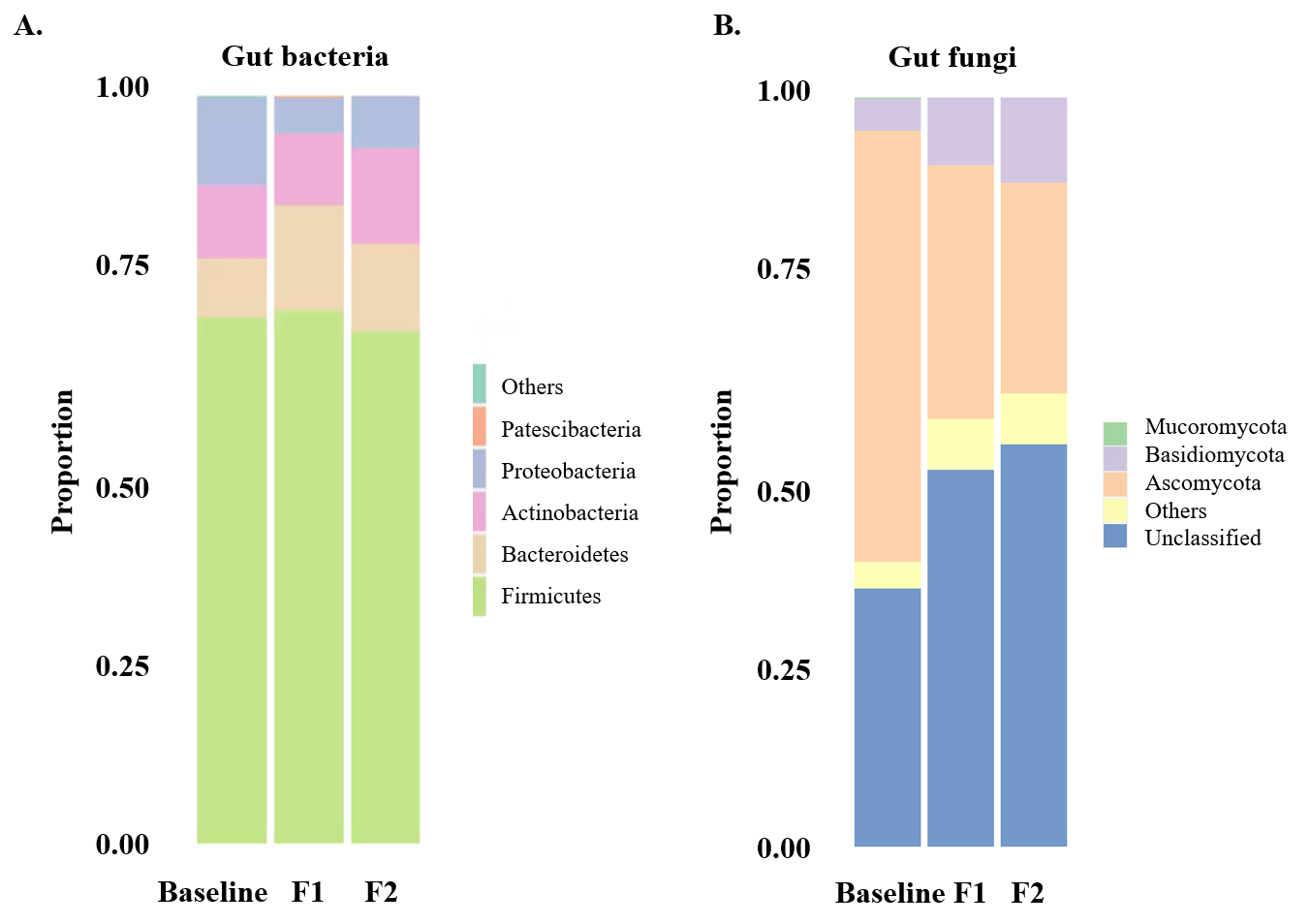
**

**Figure S3 Identification of individual genus that altered accompanying weight loss.** LDA effect size of bacterial genera that significantly altered after weight loss was calculated using LEfSe (LDA>2), a threshold of BH adjusted paired Wilcoxon p<0.01 was used for exploratory comparison between baseline and 5% weight loss among 60 paired of participants (**A**), while BH corrected paired Wilcoxon p<0.1 was applied for the confirmation analysis between baseline and 10% weight loss among 27 paired of participants (**B**). The names of genera in red colour denote weight-loss associated gut microbiota that consistently identified in both time points accompanying weight loss. LDA, linear discriminant analysis; LEfSe, linear discriminant analysis effect size; BH, Benjamini-Hochberg.


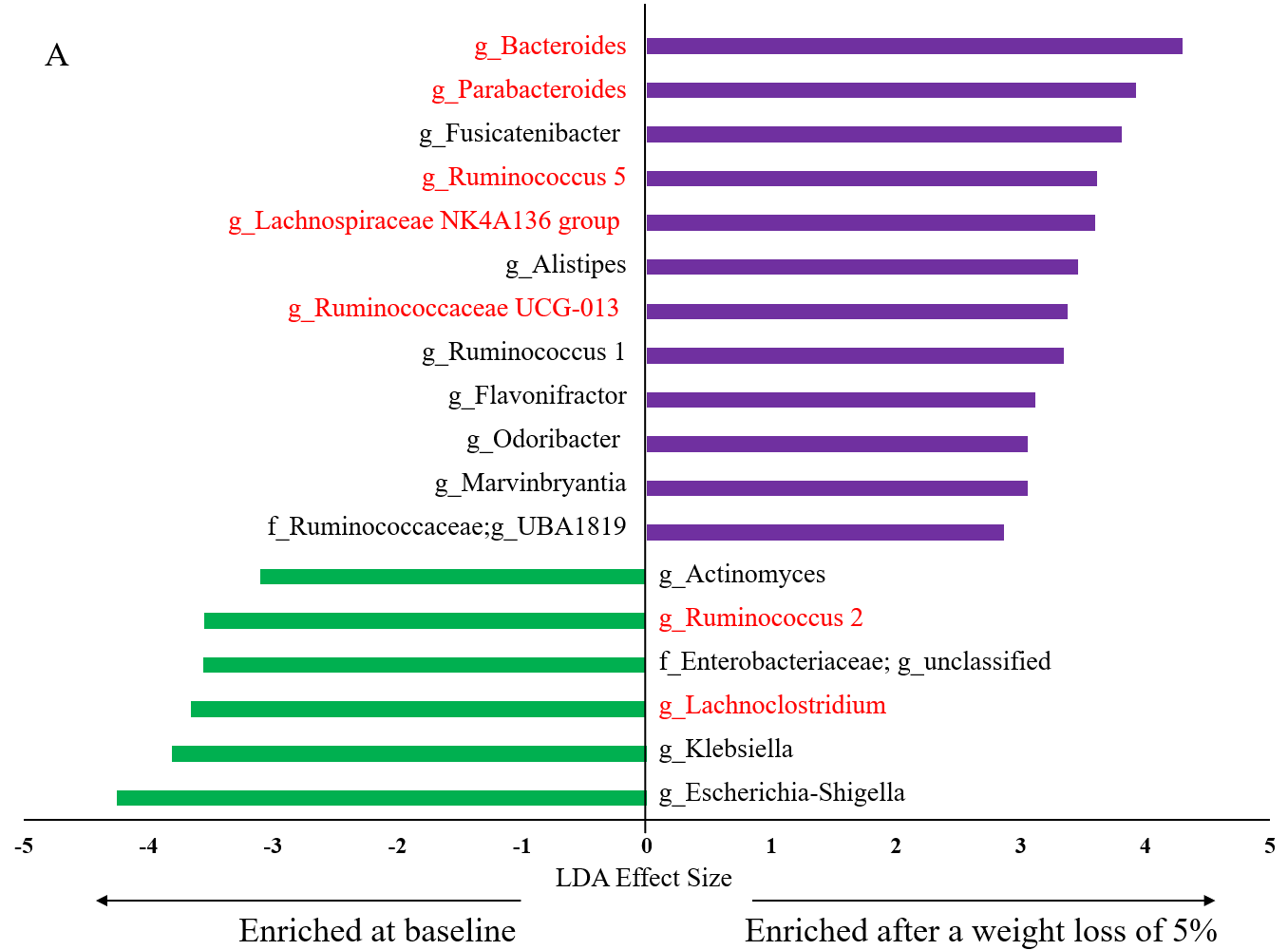


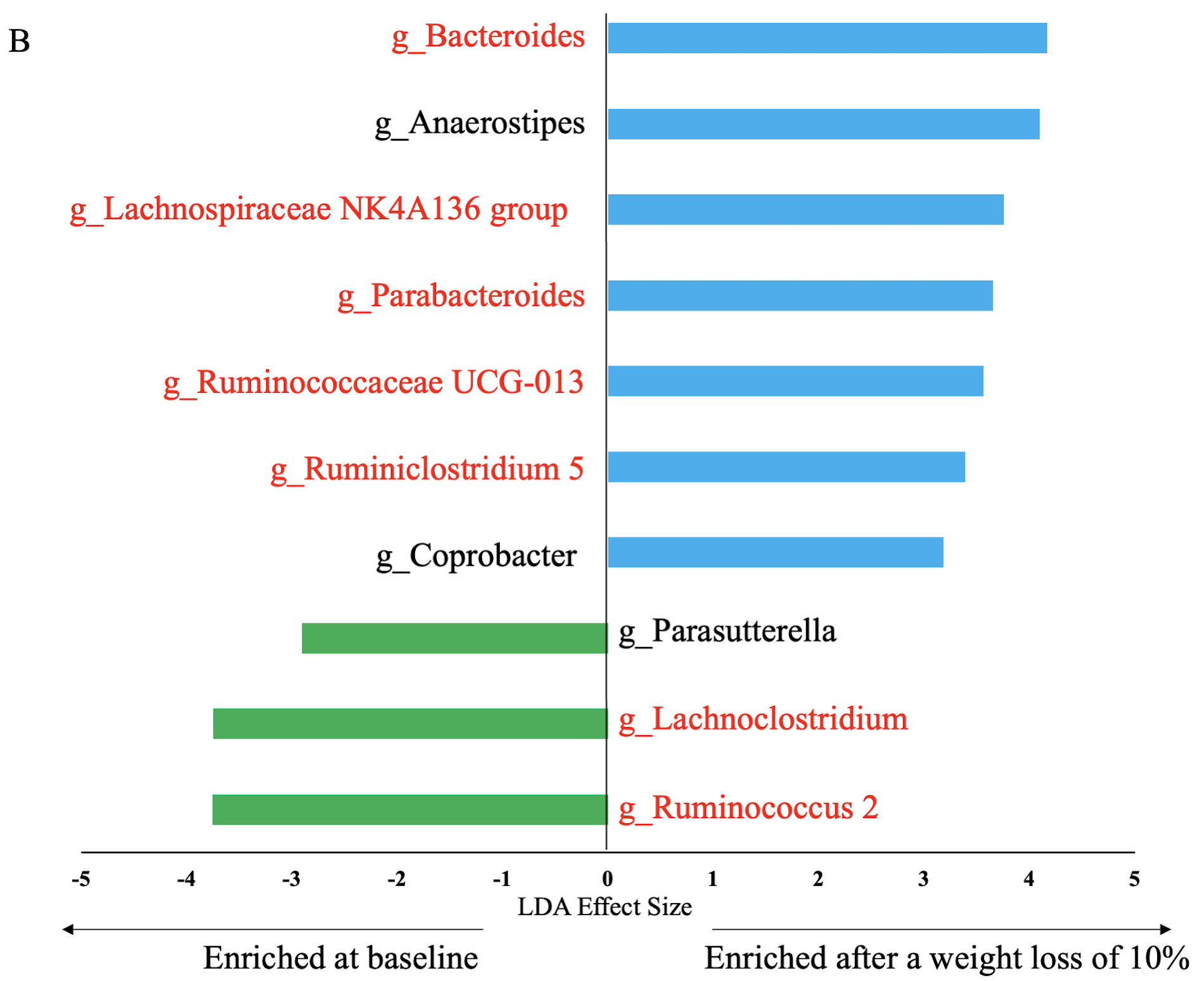


**Figure S4 Dynamics of key pathway abundance along with weight loss.** Heatmap of the relative abundance of the key gut microbiome-predicted pathways at baseline and after a weight loss of 5%. Each vertical lane corresponds to one sample.


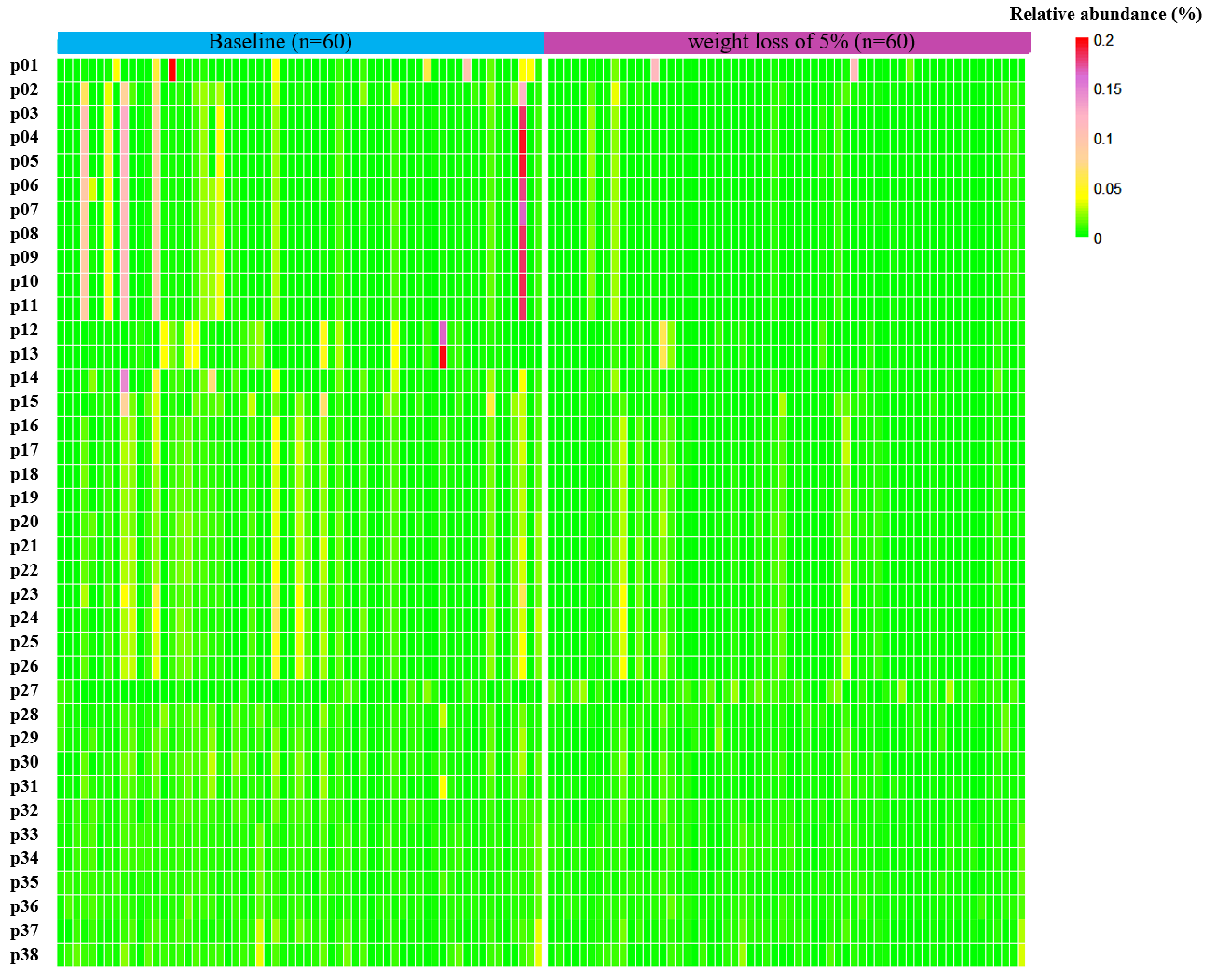


p01, glucose degradation; p02, 4-hydroxyphenylacetate degradation; p03, syringate degradation; p04, gallate degradation II; p05, methylgallate degradation; p06 catechol degradation to β-ketoadipate; p07 toluene degradation III (aerobic); p08 catechol degradation III (ortho-cleavage pathway); p09, aromatic compounds degradation via β-ketoadipate; p10 superpathway of salicylate degradation; p11, 4-methylcatechol degradation; p12, superpathway of geranylgeranyldiphosphate biosynthesis I; p13, mevalonate pathway I; p14, 3-phenylpropanoate degradation; p15, ketogluconate metabolism; p16, heme b biosynthesis I; p17 superpathway of heme b biosynthesis from uroporphyrinogen-III; p18 superpathway of heme b biosynthesis from glutamate; p19 superpathway of chorismate metabolism; p20 TCA cycle IV (2-oxoglutarate decarboxylase); p21, enterobactin biosynthesis; p22, superpathway of glycolysis, pyruvate dehydrogenase, TCA, and glyoxylate bypass; p23, L-arginine degradation II; p24, superpathway of ornithine degradation; p25 superpathway of glyoxylate bypass and TCA; p26, enterobacterial common antigen biosynthesis; p27, chondroitin sulfate degradation I; p28, inosine-5'-phosphate biosynthesis III; p29, glucose and glucose-1-phosphate degradation; p30, tRNA processing; p31, heme b biosynthesis II; p32, reductive TCA cycle I; p33, superpathway of L-tyrosine biosynthesis; p34, superpathway of L-phenylalanine biosynthesis; p35, peptidoglycan maturation;

p36, tetrapyrrole biosynthesis II; p37, superpathway of polyamine biosynthesis I; p38, early cobalt insertion.

**Supplemental table S1: 7-day Recipe**

|  | Breakfast | Lunch | Dinner |
| --- | --- | --- | --- |
| Monday | - Egg pancake with seasonal vegetable - Cucumber (raw, dressed with vinegar) - Walnut (original flavor) - Soybean milk (black beans, white kidney beans, red adzuki beans and flax seeds) | - Plain fried two melons (white gourd, loofah) - Plain fried daylily with spinach - Stewed chicken drumstick - Low-fat milk | - Fried broccoli with tomato - Plain fried green peppers with eggplant - Towel gourd and - tofu soup |
| Tuesday | - Egg (boiled) - Balsam pear (dressed with vinegar) - Walnut (original flavor) - Oatmeal blended low-fat milk | - Stewed beef with white gourd and kelp - Fried cucumber and tremella with minced garlic - Tomato and cabbage soup | - Braised tofu with scallion - Plain fried summer squash - Laver and white radish soup |
| Wednesday | - Steamed egg custard - Chinese cabbage (raw, dressed with vinegar) - Whole wheat bread - Walnut (original flavor) - Soybean milk (black beans, white kidney beans, red adzuki beans and flax seeds) | - Stir fried diced duck with celery - Garlic chrysanthemum - Low-fat milk | - Stewed winter melon and daylily - Pakchoi in sauce - Tomato and tofu soup |
| Thursday | - Whole grain and egg pancake - Cucumber (raw, dressed with vinegar) - Walnut (original flavor) - Low-fat milk | - Fried eggplant with long beans - Stir fried diced chicken with celery - Balsam pear and egg soup | - Fried tofu with lettuce - Chrysanthemum and purple cabbage in sauce - Tomato and white gourd soup |
| Friday | - Steamed egg custard - Ternip (raw, dressed with vinegar) - Steamed bread of corn - Walnut (original flavor) - Soybean milk ( black beans, white kidney beans, red adzuki beans and flax seeds) | - Stir fried chicken and cabbage - Fried towel gourd with minced garlic - Low-fat milk | - Plain fried onion with tomato - Broccoli and daylily in sauce (vinegar and garlic) - Cabbage and tofu soup |
| Saturday | - Egg (boiled) - Balsam pear (dressed with vinegar) - Walnut (original flavor) - Oatmeal blended low-fat milk | - Diced duck with lettuce - Fried romaine lettuce with minced garlic - Tremella soup | - Fried tofu with pork blood tofu - Plain fried celery and viola - Mixed mushroom soup |
| Sunday | - Fried egg - Broccoli (dressed with vinegar) - Walnut (original flavor) - Soybean milk (black beans, white kidney beans, red adzuki beans and flax seeds) | - Dumplings (stuffed with pork and cabbage) - Low-fat milk | - Fried green pepper with eggplant strips - Spinach in sauce - Tomato and tofu soup |

Note: the amount of food for each participant varies according to his/her target energy.
